# Continuous-Flow Production of Liposomes with a Millireactor under Varying Fluidic Conditions

**DOI:** 10.3390/pharmaceutics12111001

**Published:** 2020-10-22

**Authors:** Fatih Yanar, Ali Mosayyebi, Claudio Nastruzzi, Dario Carugo, Xunli Zhang

**Affiliations:** 1 Bioengineering Science Group, Faculty of Engineering and Physical Sciences, University of Southampton, Southampton SO17 1BJ, UK; F.Yanar@soton.ac.uk (F.Y.); A.Mosayyebi@soton.ac.uk (A.M.); 2 Chemical and Pharmaceutical Sciences, University of Ferrara, 35-44121 Ferrara, Italy; nas@unife.it; 3 Department of Pharmaceutics, UCL School of Pharmacy, University College London (UCL), London WC1E 6BT, UK

**Keywords:** liposome, drug delivery, continuous-flow production, millifluidic reactor, solvent exchange

## Abstract

Continuous-flow production of liposomes using microfluidic reactors has demonstrated advantages compared to batch methods, including greater control over liposome size and size distribution and reduced reliance on post-production processing steps. However, the use of microfluidic technology for the production of nanoscale vesicular systems (such as liposomes) has not been fully translated to industrial scale yet. This may be due to limitations of microfluidic-based reactors, such as low production rates, limited lifetimes, and high manufacturing costs. In this study, we investigated the potential of millimeter-scale flow reactors (or millireactors) with a serpentine-like architecture, as a scalable and cost-effective route to the production of nanoscale liposomes. The effects on liposome size of varying inlet flow rates, lipid type and concentration, storage conditions, and temperature were investigated. Liposome size (i.e., mean diameter) and size dispersity were characterised by dynamic light scattering (DLS); z-potential measurements and TEM imaging were also carried out on selected liposome batches. It was found that the lipid type and concentration, together with the inlet flow settings, had significant effects on the properties of the resultant liposome dispersion. Notably, the millifluidic reactor was able to generate liposomes with size and dispersity ranging from 54 to 272 nm, and from 0.04 to 0.52 respectively, at operating flow rates between 1 and 10 mL/min. Moreover, when compared to a batch ethanol-injection method, the millireactor generated liposomes with a more therapeutically relevant size and size dispersity.

## 1. Introduction

Liposomes are polymolecular aggregates (i.e., polymolecular assemblies) of certain amphipathic molecules, formed in aqueous solutions. They typically each consist of an aqueous core enclosed within one or more bilayers of natural or synthetic amphipathic molecules (typically glycerophospholipids, i.e., lipids that contain a glycerol-3-phosphate unit) [1,2]. Their unique architecture provides a useful platform for incorporating hydrophilic and/or hydrophobic molecules within the core and/or the bilayer, which has opened the way for the usage of liposomes as nanocarrier systems in pharmaceutical, cosmetic, and nutraceutical applications [3]. For instance, liposomes have demonstrated potential for delivering pharmaceutical actives to pathological sites within the body, with high efficacy and minimal toxicity. This is largely attributed to their nanostructure-dependent physico-chemical properties (size, surface charge, and/or hydrophilicity) together with their high biocompatibility and biodegradability, thereby reducing undesired side effects [4,5]. As a result, a number of liposome-based drug delivery systems have been developed, including conventional liposomes (mainly composed of phospholipids, with or without cholesterol), PEGylated liposomes, and ligand-targeted liposomes [6], among which Doxil^®^ and AmBisome^®^ are exemplar formulations currently in clinical use [7,8].

In the context of particulate-based drug delivery, the particle geometry (particularly its size and size distribution) plays a critical role on its performance in vivo, as it affects bio-distribution, endocytosis, clearance, targeting efficiency, drug release rate, encapsulation efficiency and stability [9,10,11,12,13]. In this regard, some of the quality control measures for food-grade or pharmaceutical-grade liposomal products involve determining the geometry and surface charge of the lipid vesicles [14]. In drug delivery applications, liposomes typically have a diameter in a range of 50 to 100 nm, which is deemed suitable for achieving vesicle’s penetration through the blood vessel wall and escaping elimination by the reticuloendothelial system [11,15,16]. The dispersity of a liposome preparation is another important parameter affecting its performance in vivo; it is often quantified from dynamic light scattering (DLS) measurements through the dispersity index, a dimensionless numerical value ranging from 0 (i.e., for a sample with a uniform particle hydrodynamic radius) to 1 (i.e., for a sample that presents a very high particle size dispersity, often characterized by multiple size populations) [11,17]. For polymer-based nanoparticles, dispersity index values smaller than 0.3 are generally considered to correspond to samples of sufficiently low size dispersity for drug delivery applications [11,18].

In principle, the formation of liposomes occurs when the phospholipid molecules encounter an aqueous environment and spontaneously arrange into a planar bilayer structure in order to minimize interactions between the hydrophobic acyl chain of the molecules and the aqueous phase. The formed planar bilayers subsequently enclose to generate a vesicular structure. For a given chemical formulation, the geometry of the resulting vesicle is primarily dependent upon the preparation process [12,19,20]. Currently, the batch methods most commonly employed for liposome production involve a series of steps, which often include (i) lipid dissolution in organic solvents; (ii) solvent evaporation; and (iii) hydration of the formed dry lipid film. However, such production methods may suffer from significant drawbacks, including limited control over process parameters to produce nanoscale vesicles with desired features, requiring additional post-production steps to achieve suitable particle dimensional properties [18,21]. These challenges become particularly critical when translating from laboratory-based synthesis to industry-scale production [18,22].

More recently, microfluidic-based devices have been employed as an alternative technique to produce liposomes with controllable size and in a continuous-flow format. In microfluidic devices, a lipid alcohol solution typically mixes with a water phase under highly controlled fluidic conditions, within microscale architectures with cross-sectional dimensions in the order of tens to hundreds of micrometers [23,24]. Microfluidic reactors with a range of channel designs, such as T or Y-shaped mixers [25,26], have been employed to produce different types of vesicular systems (including liposomes) under various flow conditions. The latter include hydrodynamic focusing [27] and droplet-based [28,29] flow regimes. Compared to traditional batch methods, microfluidic approaches have shown advantages in many aspects, including precise control over the transport of fluids, chemical species, and heat, and the potential for tuning vesicle properties on-demand [30]. However, there are still significant limitations that may hinder industrial translation of this technology; these can be mostly attributed to the small dimensional scale of the microfluidic architectures, and include high manufacturing costs, the complexity of the device’s operation, low particle production rates (up to few mg/min [31]), and the low device’s lifetime (i.e., due to potential channel blockages caused by impurities or precipitation) [32,33,34].

To address these challenges, millimeter-scale flow reactors (referred to as “millifluidic reactors” or “millireactors”) have been recently developed and employed to produce both inorganic and organic nanomaterials, at volumetric flow rates up to 18 mL/min [23,29,33,35,36,37,38,39]. Notably, scaling-up in these earlier studies enabled increased particle production capacity, while still retaining the fluidic controllability over particle’s properties. Moreover, given their larger dimensions, devices could be manufactured using cost-effective and user-friendly techniques compared to conventional microfabrication methods [29,35,40].

Although there is considerable interest in the scaling-up of liposome production [41,42,43,44,45], to the best of the authors’ knowledge, a systematic investigation of continuous-flow liposome production in a millifluidic reactor has not been carried out yet. In particular, only limited research has been performed to investigate the relationship between liposome dimensions and production/formulation-related parameters, such as inlet volumetric flow rates, lipid concentration, chemical composition, and fluid temperature. Moreover, a systematic comparison between millifluidic and batch liposome production techniques has not been fully carried out yet.

The aim of the present study was therefore to demonstrate controllable production of liposomes using an easy-to-manufacture millifluidic reactor, at a range of varying operating conditions and lipid formulations. Moreover, the stability of the produced liposomes was evaluated and production performance was compared with a commonly used batch “ethanol-injection” method.

## 2. Materials and Methods

### 2.1. Materials

Ethanol (99.9%), cholesterol (Chol, Sigma Grade ≥ 99%) from sheep’s wool, dipalmitoylphosphatidylcholine (DPPC, >99%), octadecylamine (ODA, ≥99.0%, stearylamine), and polyoxyethylene (40) stearate (PEG-40) were obtained from Sigma Aldrich, Gillingham, UK. PHOSPHOLIPON^®^90G (purified phosphatidylcholine, or PC, from soybean lecithin) was kindly provided as a gift by Phospholipid GmbH (Lipoid, Ludwigshafen, Germany). Syringe pumps (AL-1010) were purchased from World Precision Instruments (Hertfordshire, UK), and 20 mL BD-Plastipak syringes with luer lock connectors were obtained from Fisher Scientific (Loughborough, UK). Male luer lock rings, polytetrafluoroethylene (PTFE) tubing, and magnetic stirrers (UC152D) were supplied by Cole-Parmer (St. Neots, UK). The tubing that was employed to connect the outlet port of the millireactor to the collection vial was 21.5 cm long (inner diameter: 0.5 mm; outer diameter: 1.6 mm) and was purchased from Cole-Parmer (St. Neots, UK).

### 2.2. Design and Fabrication of Millireactors

**Figure 1:**
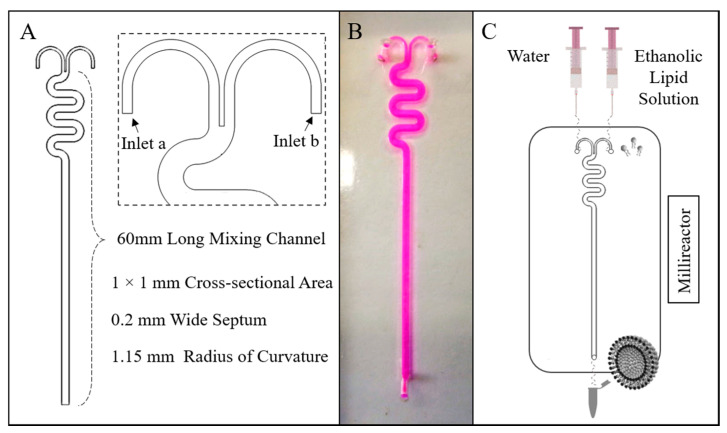
(**A**) The millireactor’s geometrical characteristics, (**B**) a top view photograph of the millireactor, and (**C**) a schematic illustration of the experimental approach for liposome production using the millireactor.

The millireactor geometry comprised two inlets with rectangular cross-sections (width: 0.4 mm, height: 1 mm) and one outlet, and a 60 mm long serpentine-like mixing channel (radius of curvature: 1.15 mm) having a square cross-sectional area of 1.0 × 1.0 mm. The curve shaped inlet channels were separated by a 0.2 mm wide septum. The geometrical layout of the millireactor and a photograph of the manufactured device (containing a coloured dye) are illustrated in Figure 1A,B, respectively.

The fabrication of the reactor was performed following a previously reported protocol, combining micromilling with replica moulding (referred to as *µ*Mi-REM) [40]. Briefly, the mould was designed in Autodesk Inventor Pro 2016 (Autodesk^®^, San Rafael, CA, USA). A negative mould was then micromilled into a block of acrylic, and epoxy resin was cast over it to obtain a positive master mould. Liquid polydimethylsiloxane (PDMS, from Sylgard^®^ 184, Dow Corning Corporation, Michigan, MI, USA) was produced by mixing a PDMS monomer with curing agent (10:1 by weight). PDMS was subsequently degassed and poured over the master mould, and cured overnight at ambient temperature to obtain a replica of the millifluidic channel architecture. Inlets/outlet ports were created through the PDMS layer using a biopsy punch (1.5 mm in diameter) with a plunger (Miltex^®^, Fischer Scientific, Loughborough, UK). Bonding of the PDMS layer with a 50 × 70 mm glass slide (Corning^®^ microscope slides, Sigma Aldrich, Gillingham, UK) was achieved via surface activation with oxygen plasma (using the TePla 300 plasma asher, PVA TePla AG, Wettenberg, Germany).

### 2.3. Liposome Production

All lipids (PC, DPPC, Chol, ODA, and PEG-40) were dissolved in ethanol. In continuous-flow liposome production by solvent exchange mechanism, the ethanolic lipid solution and water were injected separately into the two inlets of the millireactor. A schematic of the experimental set-up for the production of liposomes using the millireactor is illustrated in Figure 1C.

Different flow conditions were investigated, corresponding to variations in both the flow rate ratio (FRR) and the total flow rate (TFR). Herein, the FRR is defined as the ratio between the inlet volumetric flow rates of water and the ethanolic lipid solution, and TFR as the total volumetric flow rate (i.e., the sum of ethanol and water flow rates). Liposomes were produced either at room temperature (RT) or at 65 °C by placing the millireactor on a hot plate. In the latter case, the ethanolic lipid solution and distilled water (in separate syringes) were kept in a beaker containing water at 65 °C, prior to injection in the millifluidic device. The operational parameters (TFR, FRR, and temperature) and chemical formulations for each liposome batch are reported in Table 1. All data reported represent the mean values taken from three independent measurements of samples, with the corresponding standard deviations. Batch production of liposomes was also carried out using an ethanol injection technique [26], by manually injecting the ethanolic lipid solution in water, within a vial (4 mL) under magnetic stirring (at RT). Different volume ratios (VRs) of the aqueous phase to the ethanolic lipid solution were investigated, corresponding to FRR values of 5, 10, 25, and 50.

### 2.4. Liposome Characterization

The mean diameters, size dispersities, and zeta potentials of the produced liposome dispersions were determined by DLS technique, using a Zetasizer Nano ZS (Malvern Instruments Ltd., Malvern, UK). Dimensional and zeta potential measurements were performed at 25 °C, using polystyrene semi-micro (Fisherbrand™ (FB55147), Fisher Scientific, Loughborough, UK) cuvettes and folded capillary cell (DTS1070, Malvern Instruments Ltd., Malvern, UK) type cuvettes, respectively. Samples used for DLS analysis had a volume of 1 mL (without dilution). The viscosity values used for DLS measurements were calculated using the Zetasizer Software 7.12, by considering the effects of different FRRs and VRs on the fluid’s viscosity (see Figure S1) [46]. The dimensions of the resultant liposomes were given as the Z-average, which represents the intensity-weighted mean hydrodynamic size of the particulate suspension. The Z-average value is a widely used dimensional parameter determined using DLS according to ISO 13321 and ISO 22412, and is recommended as a robust way of quantifying and reporting the liposome’s mean size [47,48].

All measurements were performed three times per sample. The values for liposome size (Z-average, dispersity) and surface charge (zeta potential) were calculated by taking the averages of three measurements. The standard deviation was also calculated from these three measurements (using Microsoft Excel).

All graphs illustrating liposome diameter and dispersity are reported over the same y-axis range, in order to provide a comparative representation of the contributions of different formulation- and production-related parameters to liposome dimensions. However, re-scaled versions of each graph (plotted over a narrower y-axis range) are also provided in the Supplementary Materials (see Figures S5–S13).

The produced liposomes were imaged by transmission electron microscopy (TEM); 5 µL of the sample was placed on a carbon-coated grid and allowed to adsorb for 30 s, and any excess amount was removed with a filter paper (Whatman). Liposomes were negatively stained by adding 5 μL of 5% ammonium molybdate containing 1% trehalose on the grid (for 30 s), and the excess amount was again removed using a filter paper. TEM images were taken using the Tecnai T12 (FEI, Hillsboro, OR, USA).

## 3. Results and Discussion

### 3.1. Millifluidic Reactor: Design Rationale

The design of the millifluidic reactor includes two curved inlet channels, which are separated by a septum before merging into the mixing channel. This design feature was introduced to ensure that the inlet flow streams could meet parallel to each other, and differs from conventional microfluidic hydrodynamic focusing architectures where the inlet channels typically meet at an angle in the range of 30–90°. It was hypothesised that the proposed design configuration would prevent flow instabilities at the intersection between inlet channels, which could have occurred particularly at the higher flow rates investigated.

The mixing channel design had a serpentine-like architecture to enhance mixing efficiency between water and ethanol by increasing the residence time of chemical species within the device (compared to a straight channel) and by inducing advection-dominated transport, e.g., due to the formation of secondary flows within the channel’s cross-section (also known as Dean flows) [49]. The fluidic and mixing performance of the device was modelled using computational fluid dynamics (CFD) simulations (see Figure S2 in the Supplementary Materials). Values of Reynolds and Dean numbers in the mixing channel of the device, at the different TFRs investigated, are reported in Table S1. Numerical results show that increasing the TFR resulted in greater mixing efficiency, which was likely due to stronger secondary flows. At any given TFR, increasing the FRR also resulted in greater mixing efficiency [50].

### 3.2. Methodological Rationale

The first set of experiments was designed to examine the effects of TFR and FRR on the size, dispersity, and stability of liposomes. The selected values of operational parameters are comparable to those typically used in continuous-flow liposome production by solvent exchange mechanism [23]. Experiments were conducted while employing two different lipid compositions, namely, a cost-effective model lipid (PC, at 100 mM) and a formulation that is often used in commercial medicines (DPPC/Chol, at 11.2:4.8 mM) [4]. The storage stability of PC (100 mM) liposomes was also evaluated; in these experiments, liposomes were produced at a constant TFR (1 mL/min) and different FRRs (5, 10, 25, and 50). Further experiments were carried out to investigate the effects of varying the amount and/or type of the liposome constituents. These included (a) different concentrations of PC (5, 40, 100, and 200 mM) at constant TFR (1 mL/min) and FRR (5); (b) different molar ratios of PC/Chol (9:1, 8:2, 7:3, and 6:4) at constant TFR (1 mL/min) and FRR (10), corresponding to different amounts of cholesterol; and (c) different formulations of phospholipid (PC vs. DPPC) and stabilizer (Chol vs. ODA), in the presence or absence of a PEG-40 moiety (at a fixed TFR of 1 mL/min and varying FRRs). Zeta potential measurements were also performed on selected formulations, in order to evaluate the effect of lipid type on liposome surface charge. Formulations investigated had the same total concentration (16 mM) and included pure, cholesterol-containing, and octadecylamine- and cholesterol-containing PC and DPPC liposomes. Additionally, PC liposomes with a greater lipid concentration (50 mM) and DPPC liposomes containing PEG-40 (at varying concentrations) were characterised to investigate the effects of lipids and stabilizer concentration on zeta potential, respectively.

In the third set of experiments, the effect of temperature on the production of DPPC/Chol liposomes was investigated at either room temperature or 65 °C. In these experiments, the reactor was operated at varying TFRs (1, 5, and 10 mL/min) and FRRs (5, 10, 15, and 20).

Finally, a comparison between liposomes produced by millifluidics and ethanol injection (i.e., as a model for a conventional batch technique) was carried out, whereby the volume ratios (VRs) of the aqueous phase to the ethanolic lipid solution (in the batch method) were consistent with the FRR values (in the millireactor).

### 3.3. Effects of TFR and FRR on Liposome Size

**Figure 2:**
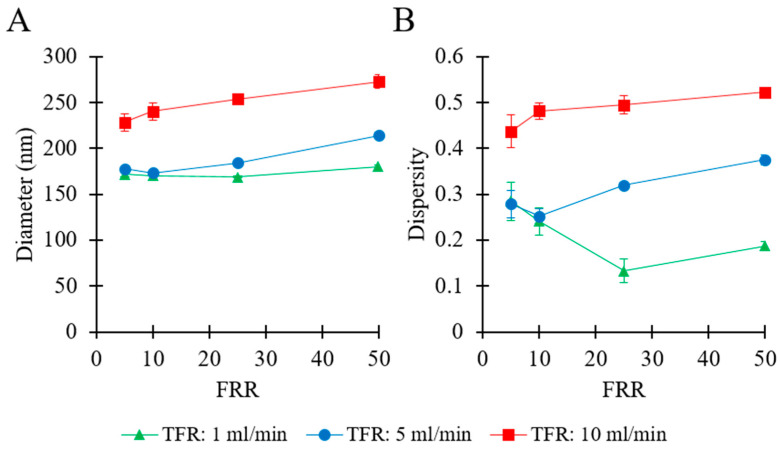
Effects of total flow rate (TFR) and flow rate ratio (FRR) on liposome Z-average size (**A**) and dispersity (**B**). Data represent the means of three measurements with corresponding standard deviations. Refer to Table 1 (batch code #1 PC to #12 PC) for the corresponding experimental conditions, lipid compositions, and numerical data of mean values and standard deviations.

The effects of fluidic parameters on liposome size and dispersity were assessed. Please refer to Table 1 (batch code #1 PC to #12 PC) for the corresponding experimental conditions and lipid composition. The experimental results are illustrated in Figure 2.

Figure 2A shows that, at a given TFR, the mean liposome diameter overall increased by increasing FRR, ranging from 169 nm (at TFR = 1 mL/min and FRR = 25) to 272 nm (at TFR = 10 mL/min and FRR = 50). At TFR = 1 mL/min, it varied in a relatively small range (from 169 to 179 nm), whilst increased from 177 nm to 214 nm at TFR = 5 mL/min, and ranged from 228 to 272 nm at TFR = 10 mL/min. At any given FRR, a greater TFR generally resulted in larger liposomes. Values of dispersity are reported in Figure 2B, and ranged from 0.13 to 0.52. At TFR = 10 mL/min, increasing the FRR from 5 to 50 resulted in an increase in dispersity from 0.43 to 0.52. At TFR = 1 mL/min, increasing FRR from 5 to 25 resulted in a decrease in dispersity (from 0.28 to 0.13), while an increase in FRR from 25 to 50 resulted in a marginal increase in dispersity (from 0.13 to 0.18). A similar trend was observed at TFR = 5 mL/min; when FRR was increased from 5 to 10 the dispersity remained almost unchanged (from 0.27 to 0.25), followed by an increase (from 0.25 to 0.37) when FRR was further increased from 10 to 50.

In summary, these results indicate that liposome size strongly depended on the imposed flow settings. The TFR was found to be directly correlated with both liposome size and size dispersity. However, diverging effects of FRR were observed at different values of TFR. At TFR = 10 mL/min, changing FRR from 5 to 50 caused an increase in both liposome size and dispersity. Conversely, at TFR = 5 mL/min or 1 mL/min, increasing the FRR did not show a significant effect at first and a subsequent tendency for an increase in liposome size. Overall, the liposome batch presenting the most suitable characteristics (for pharmaceutical applications) was obtained when operating the millireactor at TFR = 1 mL/min and FRR = 25; notably, samples produced at these operating conditions had Z-average size of 169 nm and dispersity of 0.13, and the final lipid concentration in the end-product was 3.8 mM (corresponding to 2.91 mg/mL).

**Figure 3:**
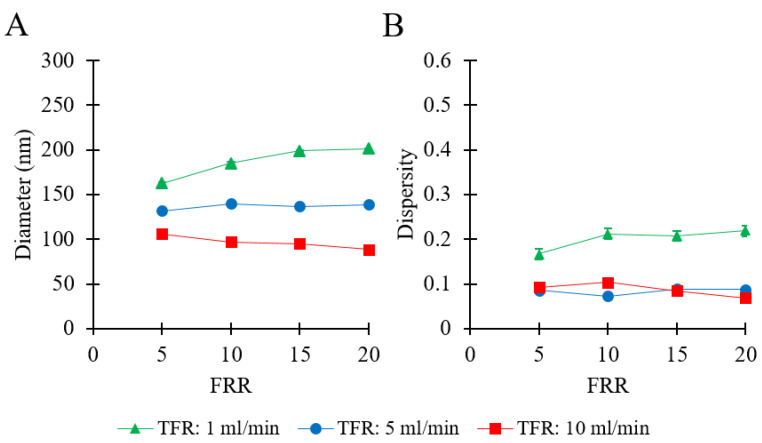
Effects of total flow rate (TFR) and flow rate ratio (FRR) on liposome Z-average size (**A**) and dispersity (**B**). Data represent the means of three measurements with corresponding standard deviations. Refer to Table 1 (batch code #27 DPPC to #38 DPPC) for the corresponding experimental conditions, lipid compositions, and numerical data of mean values and standard deviations.

To examine a formulation that is relevant to pharmaceutical applications [4], DPPC/Chol was used for experiments under similar flow conditions. Please refer to Table 1 (batch code #27 DPPC to #38 DPPC) for the corresponding experimental conditions and lipid composition. The results are shown in Figure 3.

Figure 3A shows that the largest liposomes were obtained at TFR = 1 mL/min, in a range from 162 to 201 nm (by increasing FRR from 5 to 20). At TFR = 5 mL/min, there was no significant change observed in liposome size (from 131 to 138 nm) when increasing FRR from 5 to 20. When TFR was set to 10 mL/min, increasing FRR resulted in a marginal decrease in liposome size from 105 nm to 88 nm. At any given FRR, a greater TFR resulted in smaller liposomes. The dependence of liposome size on FRR varied depending on the TFR; an increase of FRR caused either a decrease (at TFR = 10 mL/min) or an increase (at TFR = 1 mL/min) in liposome size. The values of dispersity are reported in Figure 3B and varied in a range of 0.06 to 0.21. No specific trend was observed when increasing FRR from 5 to 20, at TFR = 10 mL/min (dispersity = 0.06–0.10), TFR = 5 mL/min (dispersity = 0.07–0.08), and TFR = 1 mL/min (dispersity = 0.16–0.21). The optimal operating conditions for this formulation corresponded to TFR = 10 mL/min and FRR = 20, where the obtained liposome sample had an average size of 88 nm and dispersity of 0.07. The final total lipid concentration of the liposome samples ranged between 0.48 mg/mL (FRR = 20) and 1.67 mg/mL (FRR = 5).

The results described above (Figure 2 and Figure 3) indicate that liposome’s dimensional properties likely depended on an interplay between fluidic and chemical conditions, including TFR, FRR, and lipid concentration and type. When using the PC (100 mM) formulation, increasing TFR (at any given FRR) resulted in the production of liposomes with relatively higher diameter and size dispersity (Figure 2); conversely, when using DPPC/Chol (11.2:4.8 mM), both liposome size and size dispersity generally reduced with increasing TFR (Figure 3).

In previous studies, the liposome size was found to be almost insensitive to changes in TFR, whilst it was inversely related to FRR [23,51,52]. In these studies, liposomes were produced by the microfluidic hydrodynamic focusing (MHF) approach using microscale reactors (with a cross-section ranging between 36 to 320 μm and 10 to 320 μm in depth and width, respectively) having a cross-flow geometry, whereby vesicle formation is primarily governed by diffusion-dominated mixing between water and an organic solvent. In a previous investigation using a scaled-up MHF device with channel cross-section of 1 × 1 mm (i.e., a size that is comparable to the one used in this study), the liposome size was directly related to FRR whilst it was inversely related to TFR [23]. Additionally, liposome size was found to be inversely related to both TFR and FRR in studies where the mixing process was dominated by advection rather than diffusion, such as in zigzag-shaped microchannels [53] or semi-circular contraction-expansion array microchannels [54].

It is generally accepted that, in the liposome formation process by solvent exchange mechanism, bi-layered fragments (BFs) first form at the water–organic solvent interface, and then self-assemble into liposomes (in milliseconds) upon increased polarity of the surrounding medium [18,22]. This process is affected by the volumetric ratio between aqueous and ethanolic phases and the local lipid concentration (both depending on FRR), and by the mixing efficiency (which depends on TFR, for a given FRR). In the present study, increasing the TFR resulted in faster and more effective mixing between ethanol and water, likely due to stronger secondary flows associated with the channel’s curvature. This is confirmed by the results of numerical simulations shown in Figures S2 and S3, which show increased mixing efficiency at greater TFRs. It can be hypothesized that the rapid increase in polarity associated with a faster mixing process would in turn cause BFs to rapidly self-assemble into liposomes of a smaller diameter. Increasing the FRR also resulted in greater mixing efficiency (see numerical results reported in Figures S2 and S3), overall resulting in a decrease in liposome diameter. These predictions are consistent with the results obtained when using a lower lipid concentration (e.g., 16 mM total concentration for DPPC/Chol liposomes, Figure 3).

**Figure 4:**
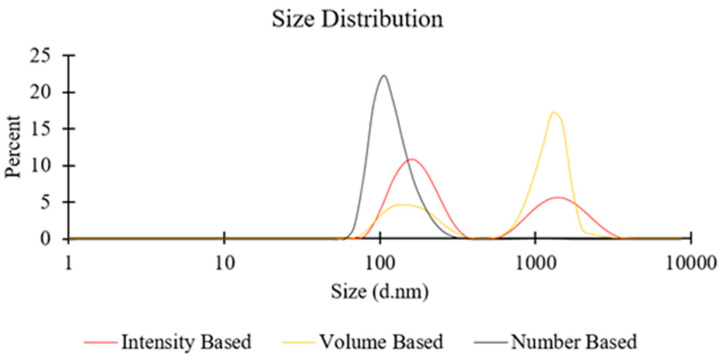
Representative size distributions (intensity, volume, and number-based) of liposome batches prepared by millifluidics, using a serpentine-shaped millireactor. Refer to Table 1 (batch code #10 PC) for the corresponding experimental conditions and lipid compositions.

On the other hand, when a greater lipid concentration was employed (e.g., 100 mM for PC liposomes; see Figure 2), the significantly larger number density of lipid molecules may have increased the possibility of BFs to collide and assemble into larger liposomal structures or other supramolecular lipid aggregates. This occurrence may become more prominent when increasing the TFR, due to the stronger secondary flows, and may also explain the observed additional peak in the DLS spectra (at approximately 1000 nm) at TFR = 10 mL/min, in both intensity-based and volume-based size distribution (see Figure 4 for representative size distribution plots). The reasons, however, remain unclear for why increasing FRR resulted in larger particles, at a TFR of 10 mL/min (Figure 2).

The liposome size distributions shown in Figure 4 were determined by taking into account the scattering intensity of each particle fraction. The peak observed at a particle size of ≈1000 nm in the intensity-based distribution potentially originated from aggregates or larger vesicles, since the particle’s scattering intensity is proportional to the square of the particle’s molecular weight. The number and volume-based distributions were instead derived from the intensity-based distributions using Mie theory. Whilst a peak at ≈1000 nm appeared in the volume-based distribution, it could not be detected in the number-based distribution; this was likely due to the fact that these larger particulate structures represent only a relatively small proportion of the overall number of particles in the dispersion [47,48].

Overall, it appears that whilst liposome size at the lower lipid concentrations is dominated by the rapidity and efficiency of mixing between ethanol and water, at the greater lipid concentrations the increased number density of lipid molecules plays a dominant role. To the best of the authors’ knowledge, this observation has not been reported previously for millifluidic-based liposome production; however, further investigations are required to fully understand the mechanisms behind vesicle formation at the fluidic conditions employed in this study.

### 3.4. Evaluation of Liposome Stability upon Storage

**Figure 5:**
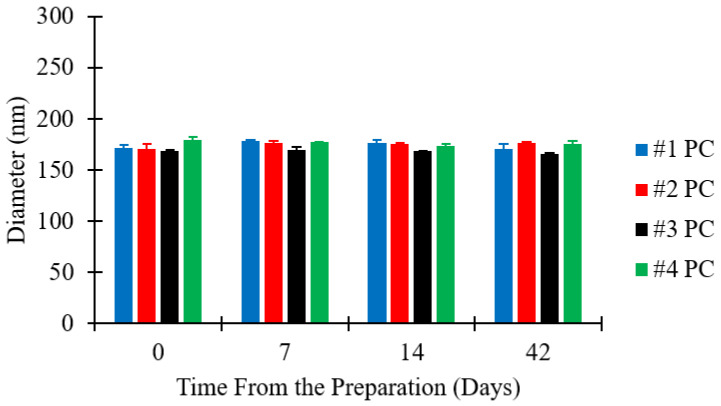
Dimensional stability of liposomes as a function of FRR. Data represent the means of three measurements with corresponding standard deviations. Refer to Table 1 (batch code #1 PC to #4 PC) for the corresponding experimental conditions, lipid compositions, and numerical data of mean values and standard deviations.

The storage stability of liposomes was assessed by analysing four different batches. Please refer to Table 1 (batch code #1 PC to #4 PC) for the corresponding experimental conditions and lipid composition. Samples were kept in glass vials at 4 °C for up to 42 days, and size measurements were taken on day 0 (just after production), day 7, day 14, and day 42. Changes in liposome size over time are reported in Figure 5.

Figure 5 shows that there was no significant change in the size of liposome batches over time. The maximum variation in liposome size was within ±4% of the initial diameter, for all liposome batches analysed. All batches prepared were thus reasonably stable over the test period of six weeks, which is consistent with previous studies reporting on the stability of liposomes produced by microfluidics [49,55]. Considering the general requirements for shelf-life of pharmaceutical products, stability measurements over longer periods may be required for certain applications.

### 3.5. The Effect of Lipid Concentration on Liposome Size

Considering the above reported results, further investigations were carried out to assess the effect of varying the concentration of PC. Please refer to Table 1 (batch codes #17 PC, #16 PC, #1 PC, #15 PC) for the corresponding experimental conditions and lipid compositions. The millifluidic device in these experiments was operated at constant flow conditions, which resulted in a final lipid concentration ranging between 0.63 and 25.3 mg/mL.

**Figure 6:**
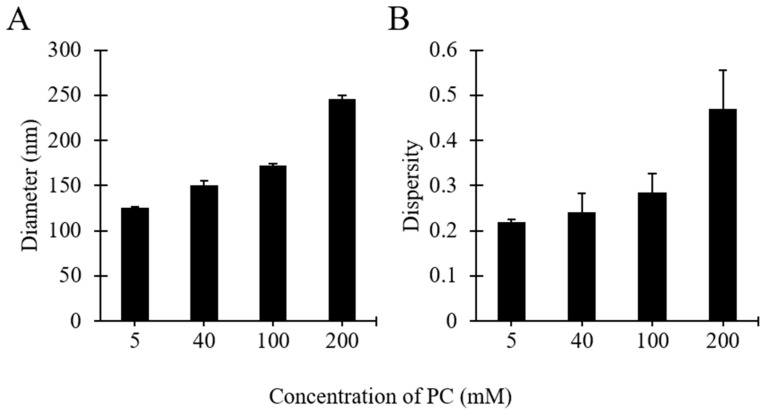
Effects of lipid concentration on liposome size (**A**) and dispersity (**B**). Data represent the means of three measurements with corresponding standard deviations. Refer to Table 1 (batch codes #17 PC, #16 PC, #1 PC, #15 PC) for the corresponding experimental conditions, lipid compositions, and numerical data of mean values and standard deviations.

As shown in Figure 6, increasing PC concentration resulted in increases of liposome diameter and size dispersity. Specifically, when increasing the initial PC concentration from 5 to 200 mM the liposome diameter increased from 125 to 245 nm (Figure 6A), and the dispersity from 0.22 to 0.46 (Figure 6B). Both these increments were more pronounced at concentrations >100 mM. These findings confirm the observations reported above (see Figure 2), and are also consistent with other studies describing the production of liposomes by microfluidic approaches [23,56]. Increasing the initial concentration of lipids likely resulted in a greater number density of lipid molecules available to form supramolecular aggregates of larger size and broader size distribution.

### 3.6. The Effect of Cholesterol on Liposome Size

Cholesterol is an important constituent of cell membranes and is widely used in liposomal formulations, as it changes the permeability and modifies the stability of liposomes [3]. It is also known to modulate the rigidity of a lipid bilayer, depending on the lipid constituents used [57]. To the best of the authors’ knowledge, a systematic investigation of the characteristics of cholesterol-containing liposomes produced by millifluidics has not been carried out yet. Therefore, in order to study the effect of cholesterol on the size of liposomes produced via millifluidics, different batches were produced by varying the amount of cholesterol relative to PC. Please refer to Table 1 (batch code #18 PC to #21 PC) for the corresponding experimental conditions and lipid composition.

**Figure 7:**
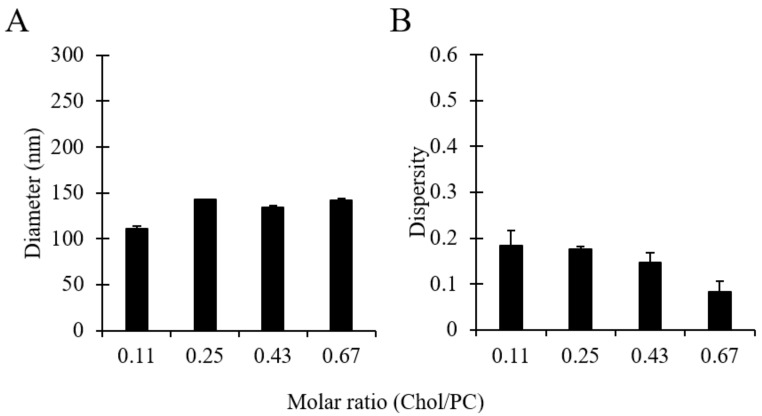
Effects of cholesterol concentration (in the molar range 1:9 to 1:1.5, at a total concentration of 16 mM) on liposome size (**A**) and dispersity (**B**). Data represent the means of three measurements with corresponding standard deviations. Refer to Table 1 (batch code #18 PC to #21 PC) for the corresponding experimental conditions, lipid compositions, and numerical data of mean values and standard deviations.

As can be observed in Figure 7, no significant differences were overall observed in liposome batches prepared with different amounts of cholesterol. Increasing the cholesterol concentration from 1:9 to 1:1.5 (in a molar range) resulted in an increase in the liposome diameter from 110 to 143 nm and a slight decrease in dispersity from 0.18 to 0.08. The marginal change in liposome size due to the addition of cholesterol could be attributed to the formation of gaps between lipid molecules resulting from the intercalation of cholesterol within the membrane bilayer, causing an expansion of the membrane, as reported in earlier investigations [58]. Moreover, the corresponding decrease in the amount of PC favoured the production of liposomes with a narrower size distribution, which is consistent with the results shown in Figure 6. Nevertheless, it is interesting to note that—in some previous studies—an increase in liposome size was observed upon increasing cholesterol concentration [58,59], with size changes depending on the lipid type and the amount of cholesterol added [60]. In summary, results demonstrated that liposomes made of different molar ratios of PC/Chol, and having a therapeutically relevant size, can be produced using the developed millifluidic device.

### 3.7. The Effects of Liposomes’ Compositions on Their Dimensional Properties

**Figure 8:**
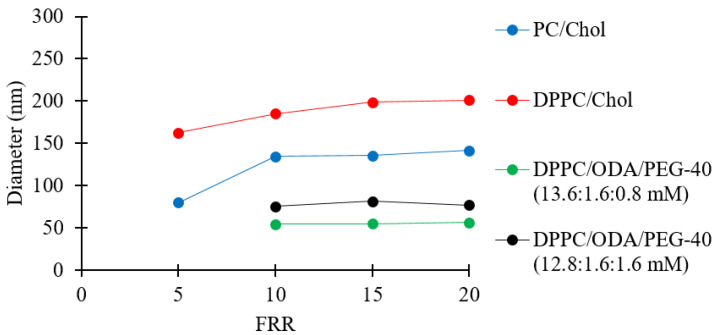
Z-average sizes of liposomes consisting of different lipid formulations. Data represent the means of three measurements with corresponding standard deviations. Refer to Table 1 (batch codes #22 PC, #20 PC, #23 PC, #24 PC, #27 DPPC, #28 DPPC, #29 DPPC, #30 DPPC #40 DPPC, #41 DPPC, #42 DPPC, #43 DPPC, #44 DPPC, #45 DPPC) for the corresponding experimental conditions, lipid compositions, and numerical data of mean values and standard deviations.

Earlier investigations of liposome production by millifluidics have predominately focused on a limited number of model lipid formulations. Since liposomes’ properties are very often dependent upon their lipid compositions [61], a further verification of the reactor’s performance was carried out by producing liposomes of different formulations comprising PC, DPPC, Chol, ODA, and PEG-40. PC (soybean) is one of the most common lipids used in liposomal formulations due to its abundance in animals and plants, and DPPC is often used in thermosensitive formulations and is often a key constituent in membrane models [62]. Cholesterol is known to increase the rigidity of a lipid bilayer [3], and PEG-40 is used to achieve steric stabilization of the liposome for applications including drug loading and release [63]. In addition, ODA (a cationic molecule) is often employed to produce positively charged liposomes in drug delivery applications [64]. Please refer to Table 1 (batch codes #22 PC, #20 PC, #23 PC, #24 PC, #27 DPPC, #28 DPPC, #29 DPPC, #30 DPPC #40 DPPC, #41 DPPC, #42 DPPC, #43 DPPC, #44 DPPC, #45 DPPC) for the corresponding experimental conditions and lipid composition. The overall results are shown in Figure 8.

Figure 8 shows that the PC/Chol and DPPC/Chol liposomes increased from 80 to 141 nm and from 162 to 201 nm, respectively, when increasing FRR from 5 to 20. The difference in size between the two formulations may have been due to the superior fluidity of the unsaturated fatty acids in the PC double layer. It has been previously reported that an increase in fluidity results in a decrease in liposome size [65]. Moreover, it has been shown that lipids with shorter chains lead to the formation of larger liposomes [66,67], which may be due to the reduced thickness of the bilayer when a shorter chain length lipid is employed, e.g., the alkyl chain lengths of DPPC (16:0) vs. PC (18:2/16:0), leading to reduced membrane bending modulus [68] and lower line tension [69]. The resulting difference in size between PC/Chol and DPPC/Chol liposomes could have been a combination of both aforementioned effects.

For the positively charged liposomes (DPPC/ODA/PEG-40), Figure 8 shows that increasing the FRR did not have a significant effect on liposome size, while simultaneously decreasing the amount of DPPC and increasing the amount of PEG-40 increased the liposome size. The observed increase in liposome size while increasing the content of PEG-40 is consistent with a previously reported investigation [70]. Please refer to Table 1 for the zeta potential values of DPPC/ODA/PEG-40 liposomes (batch code #40 DPPC to #45 DPPC).

**Figure 9:**
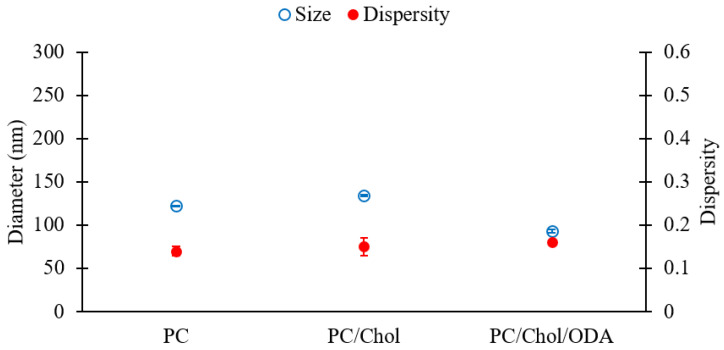
Z-average sizes (left y-axis, empty blue circles) and dispersity values (right Y-axis, filled red circles) of liposomes consisting of different lipid formulations. Data represent the means of three measurements with corresponding standard deviations. Refer to Table 1 (batch codes #13 PC, #20 PC, #25 PC) for the corresponding experimental conditions, lipid compositions, and numerical data of mean values and standard deviations.

For comparison, PC, PC/Chol, and PC/ODA/Chol liposomes were evaluated, and the results are shown in Figure 9. Please refer to Table 1 (batch codes #13 PC, #20 PC, #25 PC) for the corresponding experimental conditions and lipid compositions. Figure 9 shows that the sizes of liposomes slightly changed upon addition of cholesterol to PC (diameters of 122 and 134 nm for PC and PC/Chol liposomes, respectively) likely due to an increase in the rigidity of the membrane bilayer [57], while a slight decrease in size was observed upon the incorporation of ODA (diameter of 92 nm). No significant difference was observed in the dispersity of liposomes across formulations (Figure 9).

**Figure 10:**
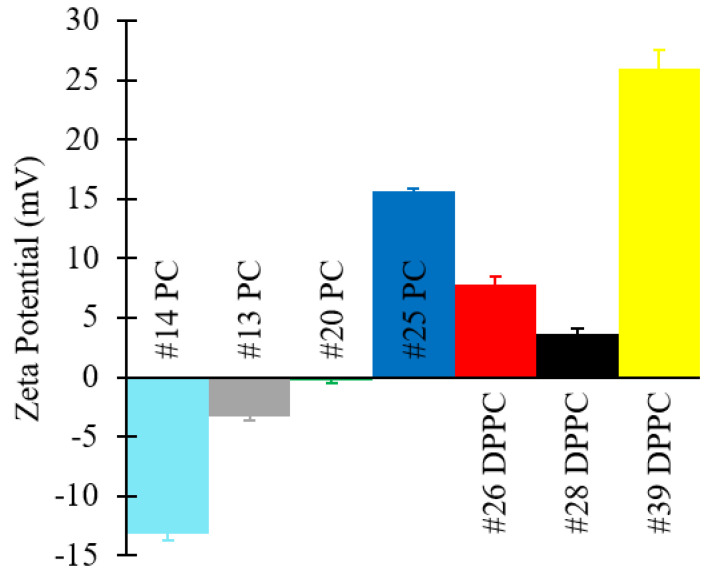
Zeta potential values of liposomes consisting of different lipid formulations. Data represent the means of three measurements with corresponding standard deviations. Refer to Table 1 (batch codes #14 PC, #13 PC, #20 PC, #25 PC, #26 DPPC, #28 DPPC, #39 DPPC) for the corresponding experimental conditions, lipid compositions, and numerical data of mean values and standard deviations.

**Figure 11:**
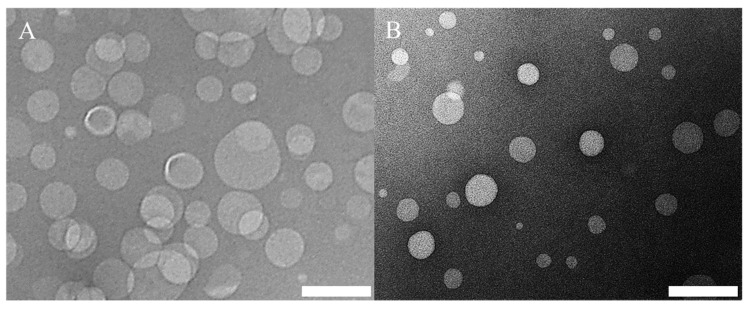
TEM images of liposome formulations of (**A**) PC/Chol and (**B**) PC/ODA/Chol. Please refer to Table 1 (batch codes #20 PC and #25 PC) for the corresponding experimental conditions and lipid compositions. Scale bars: 200 nm.

In addition, zeta potential values and TEM images of selected formulations are shown in Figure 10 and Figure 11, respectively. Please refer to Table 1 (batch codes #14 PC, #13 PC, #20 PC, #25 PC, #26 DPPC, #28 DPPC, #39 DPPC) for the corresponding experimental conditions and lipid composition.

Zeta potential values of the produced liposomes (Figure 10) show that PC formulations in the absence of ODA were negatively charged, whilst DPPC liposomes were positively charged (even in the absence of ODA). Other authors have similarly reported a slightly positive value of zeta potential for DPPC liposomes, and they have attributed this to the software used to carry out the numerical calculations [71]. As expected, the addition of ODA caused a further increase in zeta potential. Coherently with our findings (Figure 8, Figure 9 and Figure 10), it was previously reported that incorporation of ODA in liposomes resulted in a slight decrease in liposome size and an increase in surface charge [23,72]. In this study, the highest zeta potential value was observed for DPPC/ODA/PEG-40 (batch code #41 DPPC) liposomes (+45.56 mV) and the lowest for PC (batch code #14 PC) liposomes (−13.16 mV).

Representative TEM images are also illustrated in Figure 11, showing the morphologies of liposomes produced from different formulations. The size of PC/Chol liposomes (Figure 11A) was also calculated by analysing the TEM images (see Figure S4) using a custom-built particle detection software (MATLAB R2020, based on the ‘imfindcircle’ function). It was found that the value obtained from DLS (Z-average: 134.5 nm and dispersity: 0.15) was slightly larger than the one calculated from the images (number average diameter: 97.7 ± 29.8 nm and dispersity: 0.09); this small difference in diameter may have been due to differences in the size quantification method, which have already been reported and discussed in the literature [73,74].

Overall, these experiments further confirmed that liposomes of varying therapeutically-relevant formulations can be produced using the developed millifluidic reactor, resulting in end-products with a relatively uniform morphology, and of concentrations and dimensions that are relevant to pharmacological products.

### 3.8. The Effect of Production Temperature on Liposome Size

**Figure 12:**
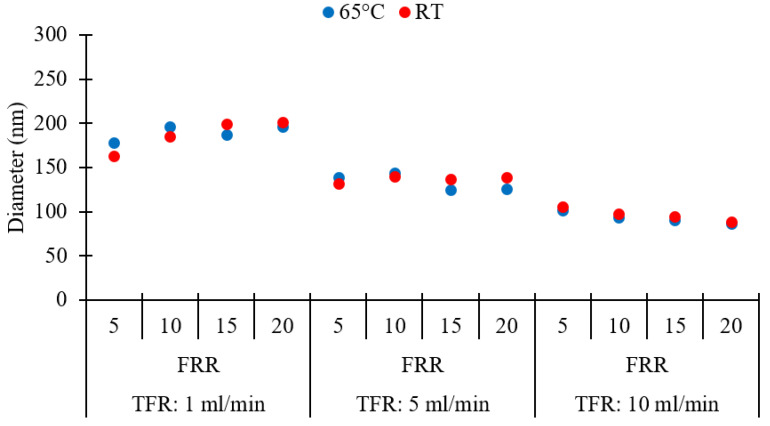
Effects of temperature on the Z-average size of different liposome batches, obtained at different values of total flow rate (TFR) and flow rate ratio (FRR). RT: room temperature. Data represent the means of three measurements with corresponding standard deviations. Refer to Table 1 (batch codes #27 DPPC to #38 DPPC, and #46 DPPC to #57 DPPC) for the corresponding experimental conditions, lipid compositions, and numerical data of mean values and standard deviations.

An evaluation of the effect of production temperature on the size of DPPC/Chol liposomes was performed by comparing liposomes produced at room temperature (RT) and 65 °C. The higher temperature was selected to be at least 10 °C above the phase transition temperature of DPPC (the reported value for the *T_m_* of pure DPPC is indeed 41.5 °C). Thus, experiments were carried out to examine whether any size change occurred when liposomes were formed above *T_m_* by millifluidic approach. Please refer to Table 1 (batch codes #27 DPPC to #38 DPPC, and #46 DPPC to #57 DPPC) for the corresponding experimental conditions and lipid composition. Results are shown in Figure 12.

It was found that increasing the temperature had insignificant effects on liposome size and dispersity (*p* > 0.05, between or within groups corresponding to 65 °C and RT). All liposome populations exhibited similar values of Z-average size (Figure 12) and dispersity (refer to Table 1), at the given TFR and FRR. A previous study found that the size of DPPC-based liposomes was larger when liposomes were prepared below the *T_m_* of the lipid but became smaller when they formed at temperatures near the phase-transition temperature; however, the liposome size was found to be less dependent on temperature, when liposome formation occurred above *T_m_* [75]. These findings also demonstrate that the millifluidic reactor can sustain temperatures up to 65 °C, without evidence of performance deterioration over the timescales of the experiment.

### 3.9. Comparison between Batch and Millifluidic-Based Liposome Production

Only a limited amount of work has been previously conducted to systematically compare millifluidic reactors with batch production methods. In this study, we compared the performance of the millifluidic reactor with that of a conventional batch ethanol-injection method, under comparable physico-chemical conditions. Batch production was carried out by first dissolving lipids in ethanol and then rapidly injecting the ethanolic lipid solution in the aqueous phase [23]. Please refer to Table 1 (batch codes #1 PC to #4 PC, and #58 PC to #61 PC) for the corresponding experimental conditions and lipid compositions.

**Figure 13:**
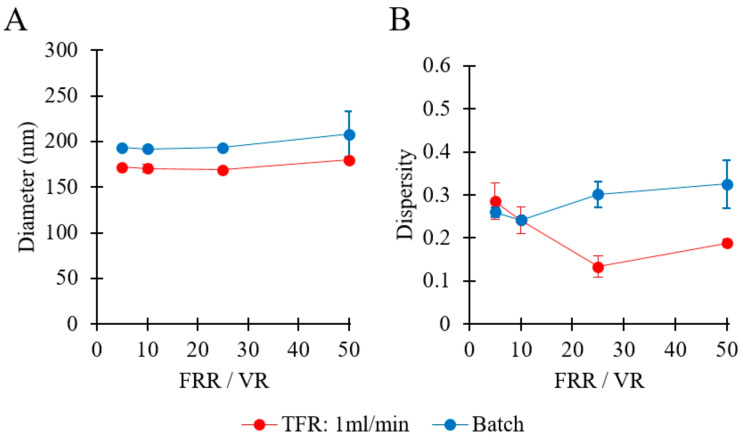
Comparison of production techniques in terms of liposome size (**A**) and dispersity (**B**). Methods of production included millifluidic-based (red) and batch ethanol injection (blue) methods. Data represent the means of three measurements with corresponding standard deviations. Refer to Table 1 (batch codes #1 PC to #4 PC, and #58 PC to #61 PC) for the corresponding experimental conditions, lipid compositions, and numerical data of mean values and standard deviations. FRR: flow rate ratio, VR: volume ratio, TFR: total flow rate.

Figure 13A compares the sizes of liposomes produced by millifluidic and batch methods. In both cases, the increase of FRR/VR caused only a very marginal change in liposome size (from 171 to 179 nm in millifluidics, and from 193 to 208 nm in batch production). Notably, liposomes produced by millifluidics were smaller than those produced by batch method, while a similar dependence of liposome diameter on FRR/VR was observed for both methods. An analogous finding was previously reported in a study comparing ethanol injection with a microfluidic hydrodynamic focusing (MHF) microscale reactor [23]. Overall, the millireactor produced smaller liposomes with a narrower size distribution when compared to the batch production method, which could be attributed to the greater controllability and rapidity of the mixing process in the millifluidic environment.

## 4. Conclusions

The use of microfluidic technology for the production of nanoscale vesicular systems, such as liposomes, has not been fully translated to the industrial scale yet. This may be due to some limitations of microfluidic-based reactors, such as low production rates, limited lifetimes, and high manufacturing costs. However, millimeter-scale flow reactors (also known as millifluidic reactors or millireactors) have the potential to achieve higher production rates and are highly suitable for scalable and low-cost manufacturing. In this study, continuous-flow production of liposomes was demonstrated using a serpentine-shaped millifluidic reactor, which could achieve production rates of up to 16.7 mg/min. The ability of the millireactor to achieve tunable production of liposomes by varying key operational parameters was demonstrated. It was found that the lipid type, along with its concentration, had a significant effect on the resultant liposome dispersions. Notably, the millireactor was able to produce DPPC/Chol liposomes with a diameter (Z-average) of approximately 100 nm and dispersity ≈0.1, when operated at a TFR of 10 mL/min with a lipid concentration of 16 mM. Additionally, the dimensions of liposomes upon storage remained largely unchanged over time. Production of liposomes at different PC concentrations, ranging from 5 to 200 mM, was also demonstrated. Overall, the reactor has proven to be suitable for the production of liposomes with a size (≈100 nm; dispersity < 0.2) that is compatible with medicinal liposomal formulations, and this was demonstrated for a spectrum of different formulations that included cholesterol, charged moieties, and PEG-40. Finally, when compared to a more conventional batch method, the serpentine-shaped millireactor generated liposomes of more therapeutically relevant size and size dispersity, demonstrating its potential for mass production of liposomes in pharmaceutical applications. Future studies may investigate millifluidic-based drug loading efficiency upon different flow conditions and a broader range of lipid concentrations, and further scaling-up of liposome production. The latter could be potentially achieved through parallelization of multiple millireactors, development of hydraulic supply units of greater capacity, or the evaluation of millifluidic devices comprising even larger architectures.

## Figures and Tables

**Table 1:** The operational parameters (flow rate ratio (FRR), total flow rate (TFR), and temperature) and chemical formulations used in each experiment.

Batch Code ^1^	Fluidic Parameters			Lipid Composition (mM) ^3^					Temperature (°C)	Size (Z-Average) (nm)	Dispersity	Z-Potential (mV)
	TFR (mL/min)	FRR	Reynolds Number ^2^	PC	DPPC	Chol	ODA	PEG-40				
#1 PC	1	5	9.79	100	-	-	-	-	RT	171.9 ± 2.7	0.285 ± 0.042	ND
#2 PC	1	10	13.17	100	-	-	-	-	RT	170.5 ± 4.6	0.241 ± 0.030	ND
#3 PC	1	25	16.18	100	-	-	-	-	RT	169.0 ± 0.8	0.133 ± 0.025	ND
#4 PC	1	50	17.30	100	-	-	-	-	RT	179.8 ± 2.4	0.188 ± 0.009	ND
#5 PC	5	5	48.95	100	-	-	-	-	RT	177.9 ± 1.8	0.279 ± 0.029	ND
#6 PC	5	10	65.83	100	-	-	-	-	RT	173.4 ± 2.0	0.252 ± 0.016	ND
#7 PC	5	25	80.88	100	-	-	-	-	RT	184.2 ± 1.5	0.320 ± 0.008	ND
#8 PC	5	50	86.52	100	-	-	-	-	RT	214.0 ± 3.3	0.375 ± 0.010	ND
#9 PC	10	5	97.90	100	-	-	-	-	RT	228.4 ± 9.5	0.437 ± 0.036	ND
#10 PC	10	10	131.66	100	-	-	-	-	RT	240.3 ± 9.2	0.481 ± 0.018	ND
#11 PC	10	25	161.75	100	-	-	-	-	RT	253.9 ± 3.0	0.494 ± 0.020	ND
#12 PC	10	50	173.04	100	-	-	-	-	RT	272.5 ± 7.4	0.522 ± 0.011	ND
#13 PC	1	10	13.17	16	-	-	-	-	RT	122.1 ± 0.5	0.145 ± 0.013	−3.4 ± 0.3
#14 PC	1	10	13.17	50	-	-	-	-	RT	ND	ND	−13.2 ± 0.5
#15 PC	1	5	9.79	200	-	-	-	-	RT	245.6 ± 4.0	0.469 ± 0.086	ND
#16 PC	1	5	9.79	40	-	-	-	-	RT	150.5 ± 5.4	0.240 ± 0.043	ND
#17 PC	1	5	9.79	5	-	-	-	-	RT	125.4 ± 1.1	0.220 ± 0.006	ND
#18 PC	1	10	13.17	14.4	-	1.6	-	-	RT	110.9 ± 2.6	0.184 ± 0.033	ND
#19 PC	1	10	13.17	12.8	-	3.2	-	-	RT	143.0 ± 0.4	0.175 ± 0.006	ND
#20 PC	1	10	13.17	11.2	-	4.8	-	-	RT	134.5 ± 1.4	0.146 ± 0.022	−0.3 ± 0.2
#21 PC	1	10	13.17	9.6	-	6.4	-	-	RT	142.3 ± 1.5	0.082 ± 0.023	ND
#22 PC	1	5	9.79	11.2	-	4.8	-	-	RT	80.0 ± 0.2	0.117 ± 0.017	ND
#23 PC	1	15	14.75	11.2	-	4.8	-	-	RT	135.8 ± 1.0	0.087 ± 0.018	ND
#24 PC	1	20	15.63	11.2	-	4.8	-	-	RT	141.5 ± 0.9	0.075 ± 0.006	ND
#25 PC	1	10	13.17	12.8	-	1.6	1.6	-	RT	92.9 ± 0.2	0.167 ± 0.006	15.6 ± 0.3
#26 DPPC	1	10	13.17	-	16	-	-	-	RT	ND	ND	7.8 ± 0.7
#27 DPPC	1	5	9.79	-	11.2	4.8	-	-	RT	162.8 ± 1.6	0.167 ± 0.046	ND
#28 DPPC	1	10	13.17	-	11.2	4.8	-	-	RT	185.2 ± 1.7	0.212 ± 0.004	3.7 ± 0.4
#29 DPPC	1	15	14.75	-	11.2	4.8	-	-	RT	198.8 ± 1.2	0.208 ± 0.012	ND
#30 DPPC	1	20	15.63	-	11.2	4.8	-	-	RT	201.1 ± 0.6	0.219 ± 0.007	ND
#31 DPPC	5	5	48.95	-	11.2	4.8	-	-	RT	131.9 ± 1.0	0.085 ± 0.028	ND
#32 DPPC	5	10	65.83	-	11.2	4.8	-	-	RT	139.7 ± 1.3	0.073 ± 0.002	ND
#33 DPPC	5	15	73.75	-	11.2	4.8	-	-	RT	136.9 ± 0.4	0.089 ± 0.016	ND
#34 DPPC	5	20	78.13	-	11.2	4.8	-	-	RT	138.6 ± 1.9	0.088 ± 0.011	ND
#35 DPPC	10	5	97.90	-	11.2	4.8	-	-	RT	105.9 ± 1.1	0.093 ± 0.017	ND
#36 DPPC	10	10	131.66	-	11.2	4.8	-	-	RT	97.3 ± 0.6	0.103 ± 0.012	ND
#37 DPPC	10	15	147.50	-	11.2	4.8	-	-	RT	94.9 ± 0.9	0.085 ± 0.011	ND
#38 DPPC	10	20	156.26	-	11.2	4.8	-	-	RT	88.4 ± 1.1	0.069 ± 0.015	ND
#39 DPPC	1	10	13.17	-	12.8	1.6	1.6		RT	ND	ND	25.9 ± 1.6
#40 DPPC	1	10	13.17	-	13.6	-	1.6	0.8	RT	54.4 ± 0.5	0.144 ± 0.020	29.8 ± 3.0
#41 DPPC	1	15	14.75	-	13.6	-	1.6	0.8	RT	54.8 ± 0.9	0.130 ± 0.007	45.6 ± 3.4
#42 DPPC	1	20	15.63	-	13.6	-	1.6	0.8	RT	56.6 ± 0.7	0.158 ± 0.013	36.3 ± 2.7
#43 DPPC	1	10	13.17	-	12.8	-	1.6	1.6	RT	75.3 ± 0.3	0.190 ± 0.016	17.4 ± 0.7
#44 DPPC	1	15	14.75	-	12.8	-	1.6	1.6	RT	81.3 ± 1.0	0.209 ± 0.010	20.8 ± 0.5
#45 DPPC	1	20	15.63	-	12.8	-	1.6	1.6	RT	77.2 ± 1.0	0.195 ± 0.007	29.8 ± 2.9
#46 DPPC	1	5	9.79	-	11.2	4.8	-	-	65	177.6 ± 2.1	0.222 ± 0.017	ND
#47 DPPC	1	10	13.17	-	11.2	4.8	-	-	65	196.1 ± 1.5	0.238 ± 0.023	ND
#48 DPPC	1	15	14.75	-	11.2	4.8	-	-	65	186.7 ± 1.9	0.154 ± 0.005	ND
#49 DPPC	1	20	15.63	-	11.2	4.8	-	-	65	195.8 ± 2.7	0.224 ± 0.017	ND
#50 DPPC	5	5	48.95	-	11.2	4.8	-	-	65	138.7 ± 2.3	0.076 ± 0.019	ND
#51 DPPC	5	10	65.83	-	11.2	4.8	-	-	65	143.5 ± 1.0	0.101 ± 0.006	ND
#52 DPPC	5	15	73.75	-	11.2	4.8	-	-	65	124.9 ± 1.3	0.094 ± 0.026	ND
#53 DPPC	5	20	78.13	-	11.2	4.8	-	-	65	125.6 ± 1.9	0.085 ± 0.015	ND
#54 DPPC	10	5	97.90	-	11.2	4.8	-	-	65	101.1 ± 0.7	0.044 ± 0.009	ND
#55 DPPC	10	10	131.66	-	11.2	4.8	-	-	65	93.9 ± 1.2	0.055 ± 0.008	ND
#56 DPPC	10	15	147.50	-	11.2	4.8	-	-	65	90.6 ± 0.4	0.057 ± 0.008	ND
#57 DPPC	10	20	156.26	-	11.2	4.8	-	-	65	86.6 ± 1.7	0.066 ± 0.008	ND
#58 PC *	-	5 *	-	100	-	-	-	-	RT	193.6 ± 2.9	0.260 ± 0.012	ND
#59 PC *	-	10 *	-	100	-	-	-	-	RT	192.1 ± 2.7	0.242 ± 0.007	ND
#60 PC *	-	25 *	-	100	-	-	-	-	RT	193.2 ± 1.7	0.302 ± 0.030	ND
#61 PC *	-	50 *	-	100	-	-	-	-	RT	208.2 ± 24.5	0.325 ± 0.056	ND

Each liposome batch is reported together with its corresponding fluidic parameters and chemical formulation, and the physico-chemical properties of the end product. Values of liposome size, dispersity, and zeta potential represent the means of three measurements with the corresponding standard deviations. Flow rate ratio (FRR) is defined as the ratio between the inlet volumetric flow rates of water and the ethanolic lipid solution, and TFR as the total volumetric flow rate (i.e., the sum of ethanol and water flow rates). ^1^ The batch code given represents the number of the produced liposome batch along with its fluidic parameters and chemical formulation, and the resultant size and dispersity values. ^2^ Reynolds number was calculated based on the volumetric ratios of water and ethanol, considering the values of TFR and FRR, and fluid’s physical properties. ^3^ Given parameters represent the amount of the phospholipid (phosphatidylcholine soybean (P90G) or dipalmitoylphosphatidylcholine (DPPC)), stabilizer (cholesterol (Chol) and/or octadecylamine (ODA)), or polyoxyethylene (40) stearate (PEG-40) in the liposome batch, reported in millimolar (mM) concentration. * The liposome batch was produced using the ethanol injection technique as a batch method; FRR values in millifluidic production correspond to the volume ratios (VRs) of water to ethanolic lipid solution in batch production. ND: not determined. RT: room Temperature.

## Supplementary Materials

The following are available online at https://www.mdpi.com/1999-4923/12/11/1001/s1, Figure S1. Data represent the viscosity values used in the DLS measurements, Figure S2. The numerically calculated relative mixing index of water and ethanol, based on the selected total flow rates (TFR) and flow rate ratios (FRR), Figure S3. Contour plots of the ethanol mass fraction, plotted over different cross-sectional surfaces from the entrance of the mixing channel until the channel’s outlet, Figure S4. (A) Shows a representative TEM image used to calculate the liposome diameter, (B) The TEM image was processed using a custom-built software (MATLAB, R2020) that detected the circular objects (i.e., liposomes) in the image and calculated their diameter, Figures S5–S13. The graphs illustrating liposome size and dispersity for the different experiments carried out in this study, reported over a *y*-axis range that has been rescaled for each data set, Table S1. Values of Reynolds number and Dean number in the mixing channel of the millifluidic device, at the different total flow rates (TFR) investigated.
