# Supplementary material for: Continuous-Flow Production of Liposomes with a Millireactor under Varying Fluidic Conditions
Source: Pharmaceutics. 2020 Oct 22;12(11):1001. doi: 10.3390/pharmaceutics12111001 (PMC7690435; doi:10.3390/pharmaceutics12111001)
Supplement: Supplementary file 1 [file pharmaceutics-12-01001-s001.pdf]

pharmaceutics

Supplementary Materials: Continuous-Flow Production of Liposomes with a Millireactor under Varying Fluidic Conditions

Fatih Yanar, Ali Mosayyebi, Claudio Nastruzzi, Dario Carugo, Xunli Zhang

S1. Calculation of Reynolds number (Re) and Dean number (De)

Table S1 summarises the values of Reynolds number (Re) and Dean number (De) in the mixing channel of the millifluidic reactor, calculated at different total flow rates (TFR).

Table S1. Values of Reynolds number and Dean number in the mixing channel of the millifluidic device, at the different total flow rates (TFR) investigated.

TFR (ml/min)	Reynolds number	Dean Number
1	13.1	8.6
5	65.8	43.4
10	131.6	86.8

The Reynolds number (Re) and Dean number (De) were calculated from $Re = \rho VD/\mu$ and $De = ((D/2r)^{0.5})Re$, respectively, where ρ is the fluid density, V is the mean velocity of the fluid, D is the channel hydraulic diameter, μ is the fluid dynamic viscosity, and r is the radius of curvature of the mixing channel centerline. The density of the ethanol-water mixture was calculated based on the FRR, and the dynamic viscosity was obtained from the viscosity values reported in Figure S1. Values were calculated considering a flow rate ratio (FRR) of 10.

S2. Viscosity of the ethanol/water solution

Figure S1 shows the dynamic viscosity of the ethanol/water mixture, at varying amounts of water, determined using the Zetasizer software.

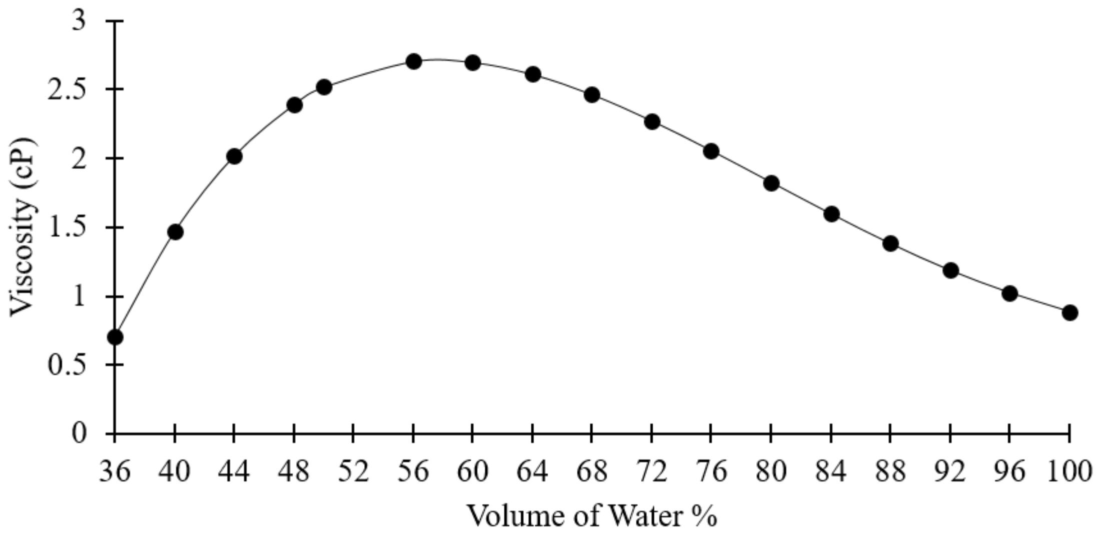

Figure S1. Data represent the viscosity values used in the DLS measurements (in cP). Depending on the flow rate ratio (FRR) used, the volume ratio of ethanol and water was calculated theoretically and the viscosity values were extrapolated using the software (Zetasizer) provided by Malvern.

The concentration of the water-ethanol mixture was calculated theoretically by considering the varied amount of water at different FRRs, and was input in the Zetasizer software to obtain the viscosity values reported in Fig. S1. The reported values were in turn input in the DLS software for determination of liposome size and dispersity.

S3. Numerical simulation of the transport of fluids and chemical species within the millifluidic device

The transport of fluid and chemical species (ethanol and water) within the millifluidic device was characterised using computational fluid dynamics (CFD) simulations. The geometry of the millireactor was designed using Inventor Pro 2016 (Autodesk®, USA), and geometrical meshing was performed using ICEM CFD 17.0 (Ansys Inc., USA). A total number of 1’661’007 mesh volumes (selected mesh size of 0.05 mm) of tetrahedral shape was employed. Ansys® Fluent 17.0 was employed to solve for mass and momentum conservation equations (i.e., Navier-Stokes), and for advection-diffusion equations. Selected TFR and FRR values, corresponding to experimental conditions tested, were simulated numerically. Boundary conditions were set as: constant mass flow at the inlet cross-sections, atmospheric pressure at the outlet, and no-slip boundary condition at the channel inner walls. The diffusion coefficient of ethanol in water was set to 1×10^{-9} m^2/s [1], and fluids were assumed incompressible and Newtonian.

In order to quantify the mixing performance of the device, the relative mixing index (RMI) was calculated for each simulated flow condition. RMI was defined as the ratio of the standard deviation of ethanol mass fraction over the outlet cross-section to the standard deviation of ethanol mass fraction in the unmixed state (just after the junction between inlets), following an approach previously reported in the literature [2].

Figure S2 shows the calculated values of relative mixing index at different values of flow rate ratio and total flow rate, demonstrating an increase in mixing efficiency with increasing both TFR and FRR.

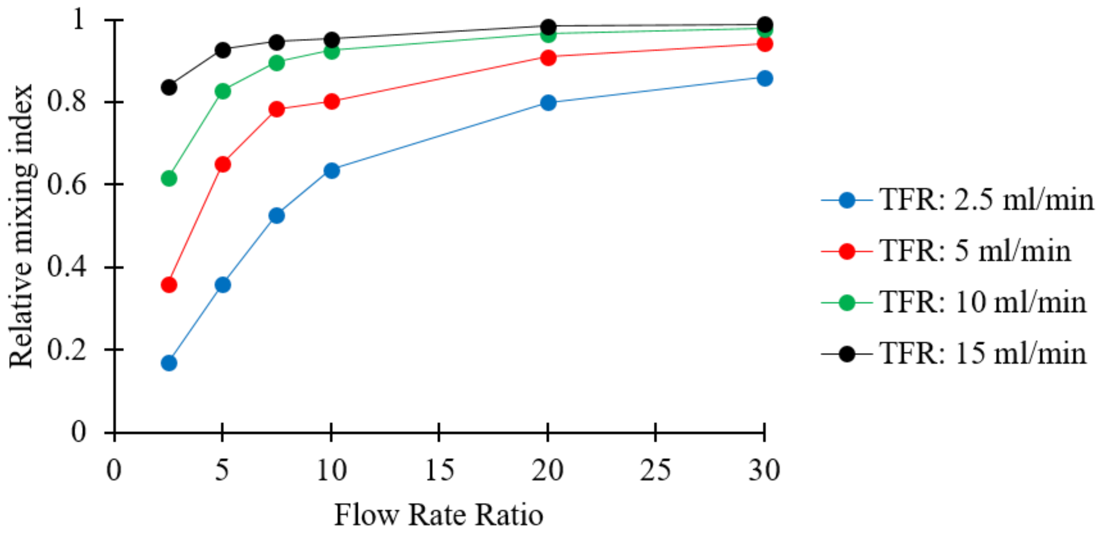

Figure S2. The numerically calculated relative mixing index of water and ethanol, based on the selected total flow rates (TFR) and flow rate ratios (FRR).

Figure S3 instead shows contours of ethanol mass fraction plotted over selected cross-sectional surfaces along the mixing channel of the millifluidic device, at varying operational TFRs and FRRs.

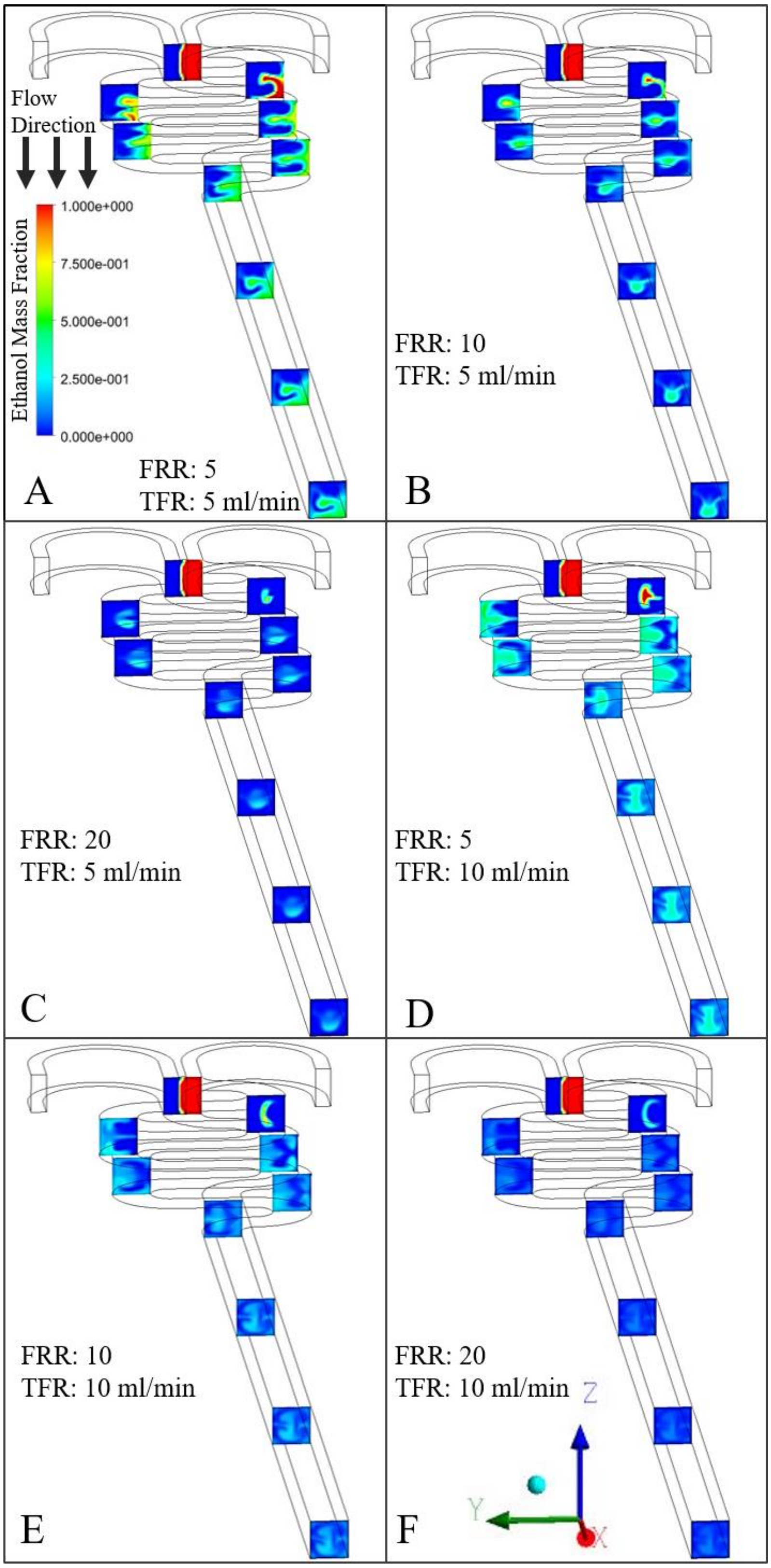

Figure S3. Contour plots of the ethanol mass fraction, plotted over different cross-sectional surfaces from the entrance of the mixing channel until the channel's outlet. The red and blue colours represent the ethanol and water phases, respectively. Mixing between the two phases along the millifluidic reactor can be appreciated. (**A**) TFR = 5 mL/min, FRR = 5. (**B**) TFR = 5 mL/min, FRR = 10. (**C**) TFR = 5 ml/min, FRR = 20. (**D**) TFR = 10 mL/min, FRR = 5. (**E**) TFR = 10 mL/min, FRR = 10. (**F**) TFR = 10 mL/min, FRR = 20. TFR: Total flow rate, and FRR: flow rate ratio (aqueous/ethanolic lipid solution).

S4. Quantification of liposome diameter from TEM images

Figure S4A illustrates a representative TEM image of liposomes (PC/Chol 11.2:3.8 mM) produced by millifluidics. Figure S4B shows the outcome of the particle detection script (written in MATLAB) employed to detect circular objects (i.e. liposomes) in the TEM images and quantify their diameter.

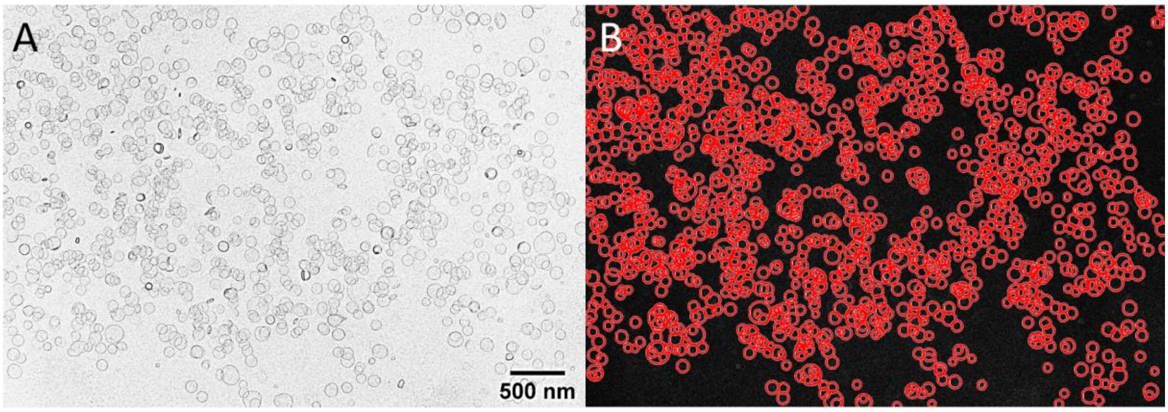

Figure S4. (**A**) Shows a representative TEM image used to calculate the liposome diameter. Liposomes comprised of PC/Chol (11.2:3.8 mM) and were produced using the millifluidic reactor, at total flow rate (TFR) = 1 mL/min and flow rate ratio (FRR, aqueous phase/ethanolic lipid solution) = 10. (**B**) The TEM image was processed using a custom-built software (MATLAB, R2020) that detected the circular objects (i.e. liposomes) in the image and calculated their diameter.

The image-based method of calculation of the mean diameter was based on a determination of the number average diameter of detected circles in the TEM images. The method implemented by MATLAB uses the function "imfindcircles", which detects circles in an image *via* the Hough transform. The method can also discriminate between overlapping circles, and provides the diameter of each individual circle. The average liposome diameter (97.7 ± 29.8 nm) was calculated by dividing the sum of the diameter values of each circle to the number of circles detected. The dispersity (0.09) was calculated by taking the square of the standard deviation divided by the mean diameter [3].

S5. Liposome size and dispersity: graphs with re-scaled *y*-axis

Values of liposome diameter and dispersity obtained at varying production- and formulation-related conditions are reported below. Figures correspond to the ones included in the main manuscript body, with the difference that the *y*-axis has been re-scaled for each set of plotted data.

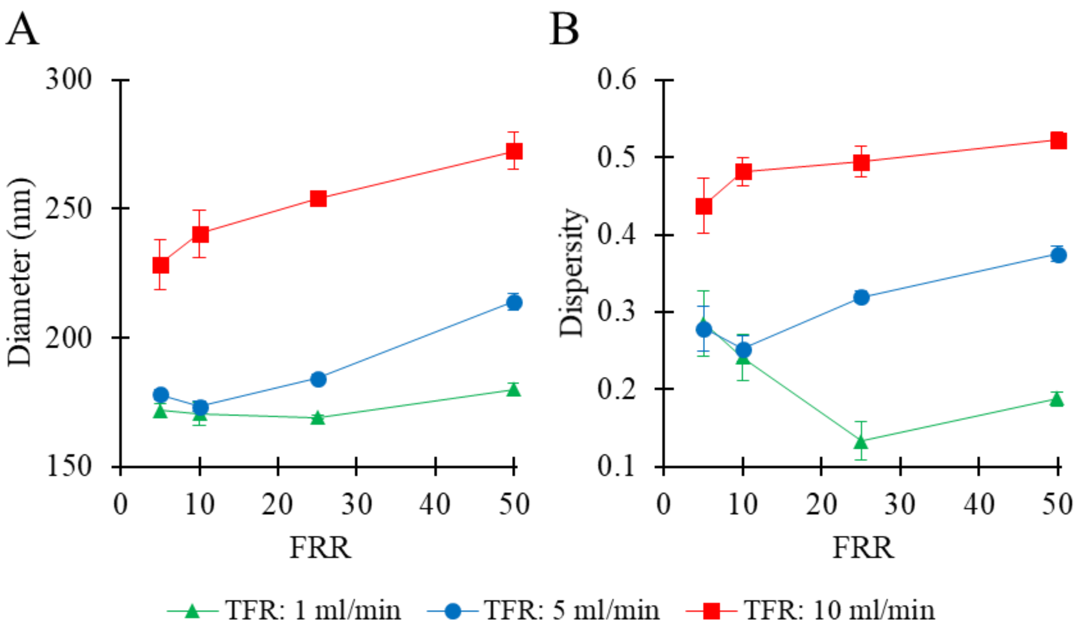

Figure S5. Effects of total flow rate (TFR) and flow rate ratio (FRR) on liposome Z-average size (A) and dispersity (B). Data represent the mean of three measurements with corresponding standard deviation. Refer to Table 1 (batch code #1 PC to #12 PC) for the corresponding experimental conditions, lipid composition, and numerical data of mean values and standard deviation.

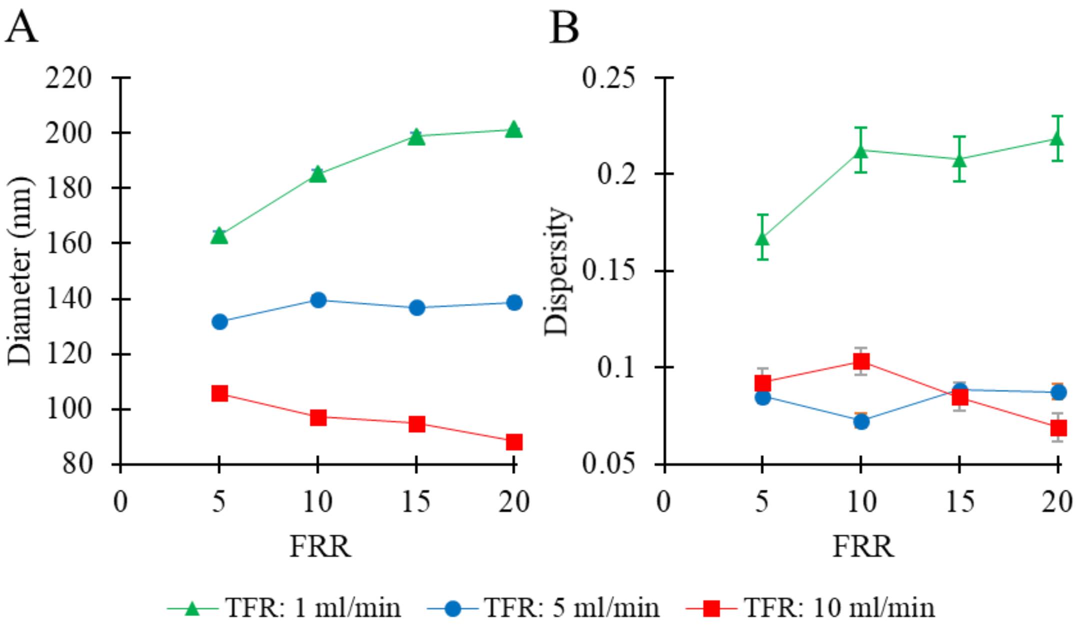

Figure S6. Effects of total flow rate (TFR) and flow rate ratio (FRR) on liposome Z-average size (**A**) and dispersity (**B**). Data represent the mean of three measurements with corresponding standard deviation. Refer to Table 1 (batch code #27 DPPC to #38 DPPC) for the corresponding experimental conditions, lipid composition, and numerical data of mean values and standard deviation.

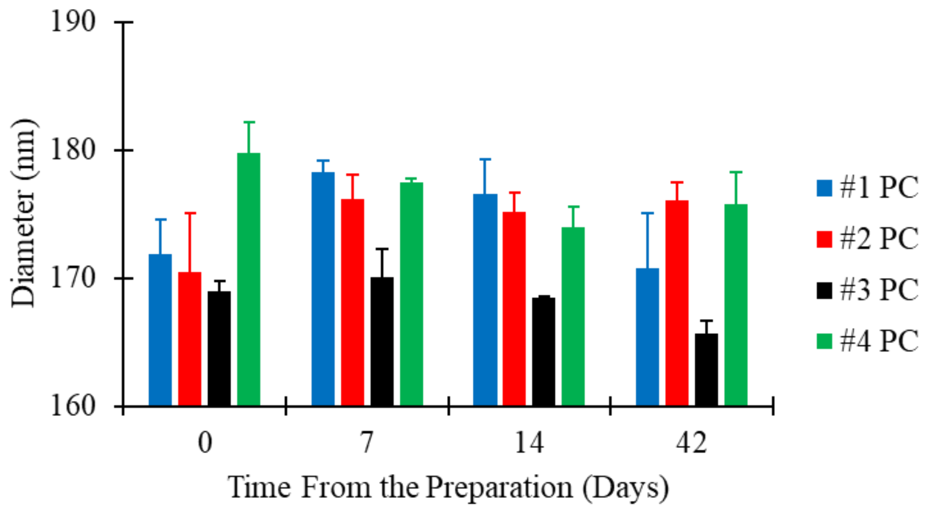

Figure S7. Dimensional stability of liposomes as a function of FRR. Data represent the mean of three measurements with corresponding standard deviation. Refer to Table 1 (batch code #1 PC to #4 PC) for the corresponding experimental conditions, lipid composition, and numerical data of mean values and standard deviation.

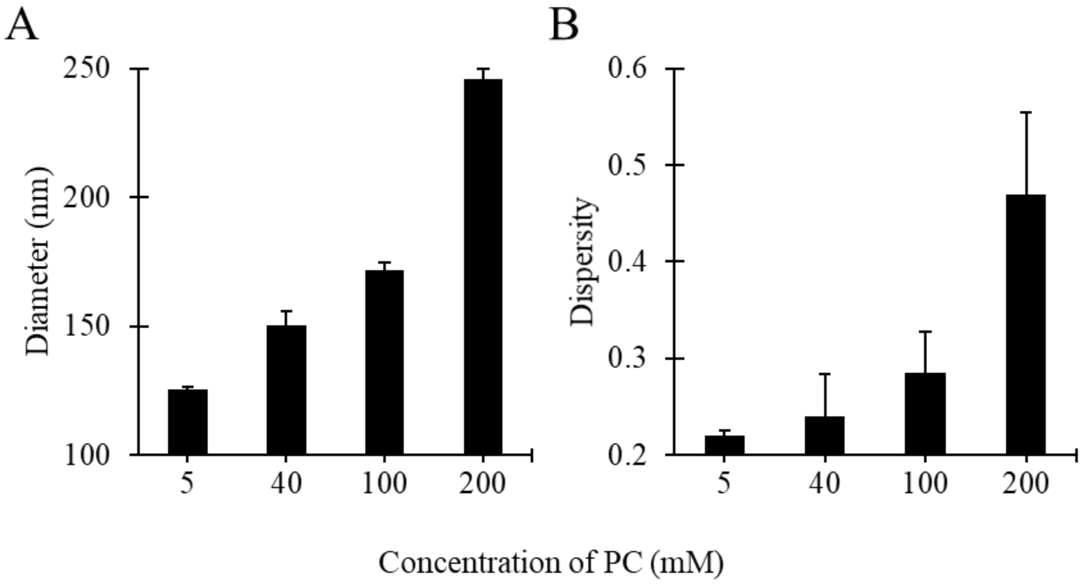

Figure S8. Effect of lipid concentration on liposome size (**A**) and dispersity (**B**). Data represent the mean of three measurements with corresponding standard deviation. Refer to Table 1 (batch codes #17 PC, #16 PC, #1 PC, #15 PC) for the corresponding experimental conditions, lipid composition, and numerical data of mean values and standard deviation.

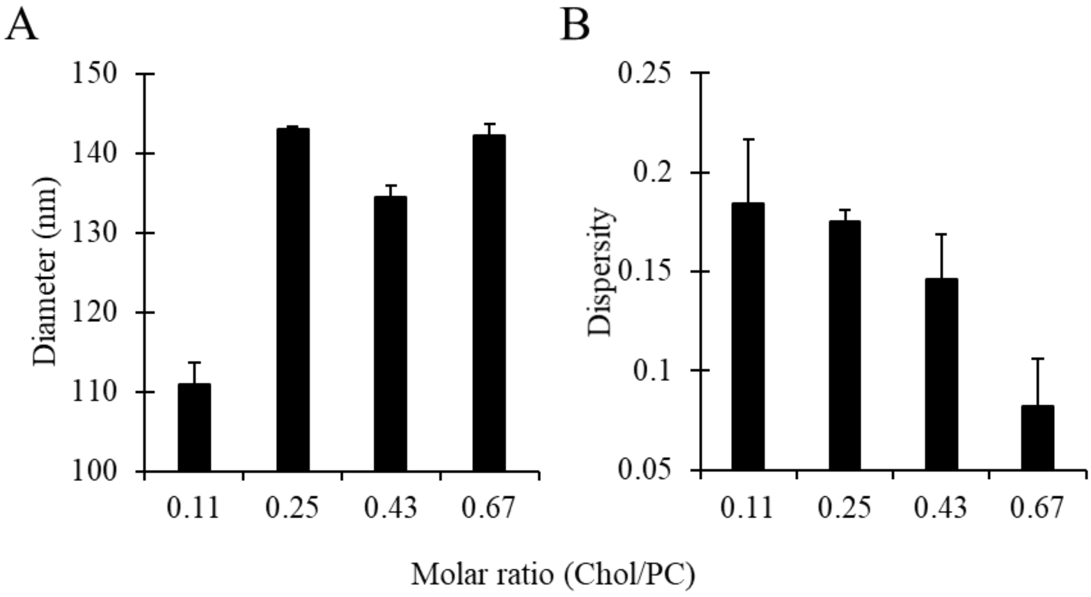

Figure S9. Effect of cholesterol concentration (in the molar range 1:9 to 1:1.5, with a total concentration of 16 mM) on liposome size (**A**) and dispersity (**B**). Data represent the mean of three measurements with corresponding standard deviation. Refer to Table 1 (batch code #18 PC to #21 PC) for the corresponding experimental conditions, lipid composition, and numerical data of mean values and standard deviation.

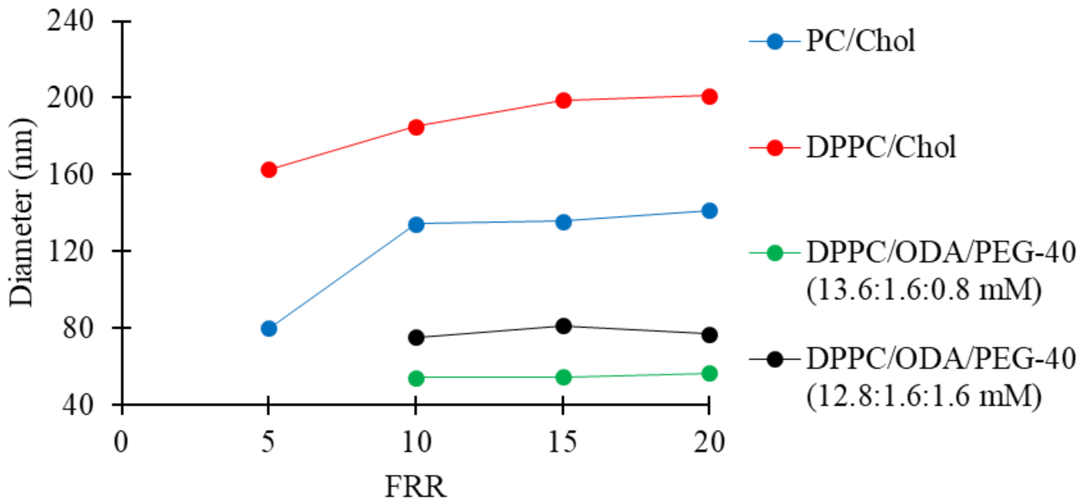

Figure S10. Z-average size of liposomes consisting of different lipid formulations. Data represent the mean of three measurements with corresponding standard deviation. Refer to Table 1 (batch codes #22 PC, #20 PC, #23 PC, #24 PC, #27 DPPC, #28 DPPC, #29 DPPC, #30 DPPC #40 DPPC, #41 DPPC, #42 DPPC, #43 DPPC, #44 DPPC, #45 DPPC) for the corresponding experimental conditions, lipid composition, and numerical data of mean values and standard deviation.

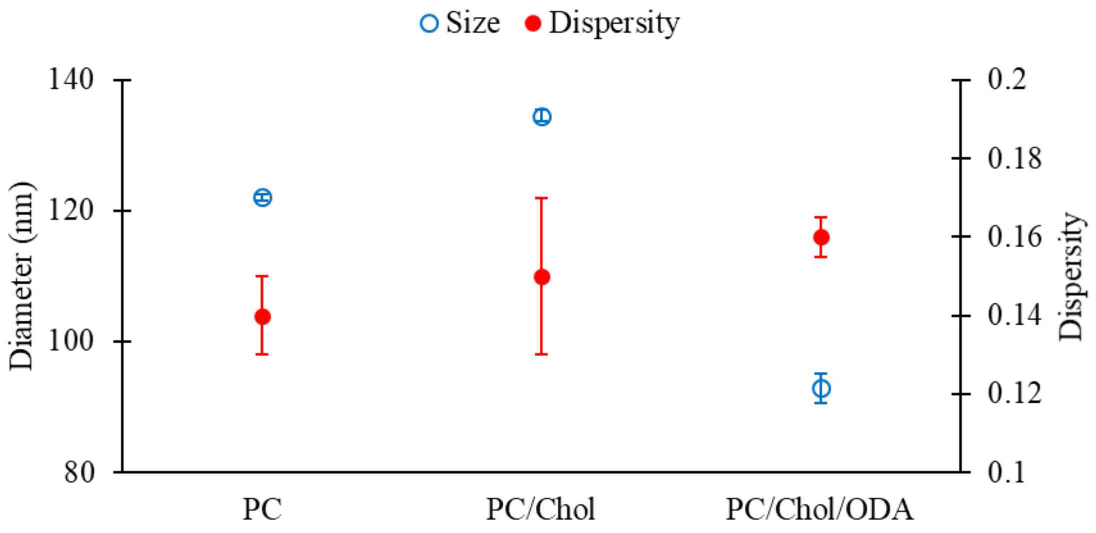

Figure S11. Z-average size (left Y-axis, empty blue circles), and dispersity (right Y-axis, filled red circles) of liposomes consisting of different lipid formulations. Data represent the mean of three measurements with corresponding standard deviation. Refer to Table 1 (batch codes #13 PC, #20 PC, #25 PC) for the corresponding experimental conditions, lipid composition, and numerical data of mean values and standard deviation.

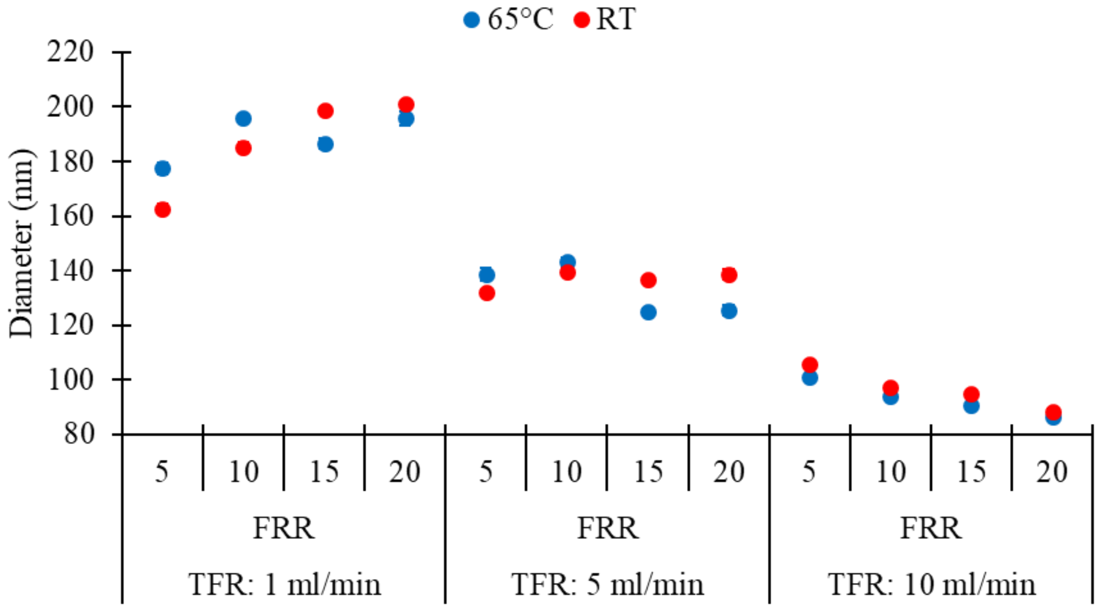

Figure S12. Effect of temperature on Z-average of different liposome batches, obtained at different values of total flow rate (TFR) and flow rate ratio (FRR). RT: Room temperature. Data represent the mean of three measurements with corresponding standard deviation. Refer to Table 1 (batch codes #27 DPPC to #38 DPPC, and #46 DPPC to #57 DPPC) for the corresponding experimental conditions, lipid composition, and numerical data of mean values and standard deviation.

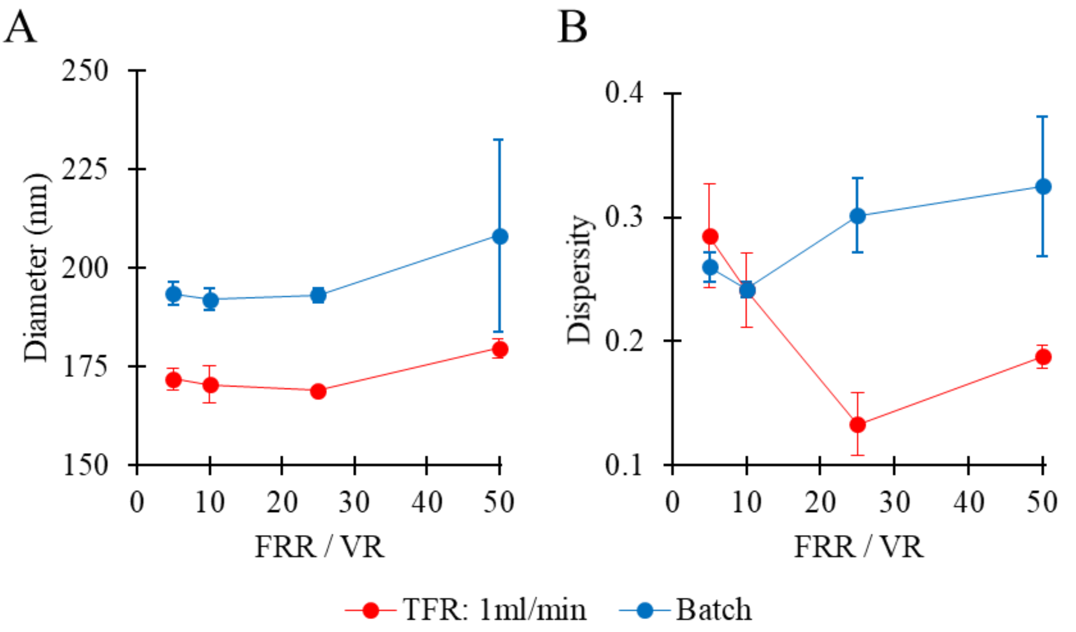

Figure S13. Comparison of production techniques in terms of liposome size (**A**) and dispersity (**B**). Methods of production included millifluidic-based (red) and batch ethanol injection (blue) methods. Data represent the mean of three measurements with corresponding standard deviation. Refer to Table 1 (batch codes #1 PC to #4 PC, and #58 PC to #61 PC) for the corresponding experimental conditions, lipid composition, and numerical data of mean values and standard deviation. FRR: flow rate ratio, VR: volume ratio, TFR: total flow rate.

References

1. Hills, E.E., et al., *Diffusion coefficients in ethanol and in water at 298 K: Linear free energy relationships.* Fluid Phase Equilibria, 2011. **303**(1): p. 45-55.
2. Hashmi, A. and J. Xu, *On the quantification of mixing in microfluidics.* Journal of laboratory automation, 2014. **19**(5): p. 488-491.
3. Composix, N., *Nano Composix's Guide To Dynamic Light Scattering Measurement and Analysis.* NanoComposix, San Diego, Calif, USA, 2012.
